# Analysis of the trend of notifiable sexually transmitted infections in China between 2006–22

**DOI:** 10.7189/jogh.15.04175

**Published:** 2025-06-27

**Authors:** Qingsong Xu, Xiyu Zhang, Tianshuo Zhao, Xianming Cai, Sihui Zhang, Mingting Wang, Qing-Bin Lu, Fuqiang Cui

**Affiliations:** 1 Department of Epidemiology and Biostatistics, Peking University, School of Public Health, Beijing, China; 2 Vaccine Research Centre, School of Public Health, Peking University, Beijing, China; 3 Centre for Infectious Diseases and Policy Research and Global Health and Infectious Diseases Group, Peking University, Beijing, China; 4 Key Laboratory of Epidemiology of Major Diseases, Peking University, Ministry of Education, Beijing, China; 5 Beijing Key Laboratory of Toxicological Research and Risk Assessment for Food Safety, Beijing, China

## Abstract

**Background:**

AIDS, gonorrhoea, and syphilis are the most common sexually transmitted infections, causing a heavy burden in China and worldwide. We analysed the long-term spatial and temporal trends of AIDS, gonorrhoea, and syphilis in the Chinese mainland between 2006–22.

**Methods:**

We extracted the data on incidence and mortality of AIDS, gonorrhoea, and syphilis from the annual surveillance reports of China Information System for Disease Control and Prevention between 2006–22, which was developed by the Chinese Centre for Disease Control and Prevention and contains all notifiable diseases data. We used locally weighted regression to draw the trend curve and joinpoint regression analysis to calculate the average annual percentage change. We used the Bayesian structured time series model to evaluate the seasonal trends of sexually transmitted infections by province and the Moran I statistic to analyse the spatial clustering of sexually transmitted infections, detecting hot spots, cold spots, and spatial outliers.

**Results:**

The incidence and mortality of sexually transmitted infections in the Chinese mainland were on the rise, especially in the group aged 15–24 years and the elderly aged ≥60. The incidence of AIDS and gonorrhoea in males was higher than in females. The mortality of the elderly aged ≥60 increased rapidly. The incidence of gonorrhoea and syphilis had a seasonal distribution. The high incidence of gonorrhoea was concentrated from May–November, and syphilis was concentrated from March–August. Between 2006–22, there was a trend of spatial clustering in sexually transmitted infections, forming high-high clusters and low-low clusters with clear boundaries.

**Conclusions:**

Schools could incorporate sexual health education into compulsory courses and the government may consider incorporating sexually transmitted infections rapid testing into routine chronic disease management. The interventions are needed to prevent the incidence among high-risk populations before summer, and focus on the Southwest, East China, and West China, where the incidence and case-fatality rates were high.

Sexually transmitted infection (STI) is an infection caused by a pathogen through sexual contact, and develops into sexually transmitted disease when a recognisable disease state occurs [1]. STIs cause acute urogenital conditions such as cervicitis, urethritis, vaginitis, and genital ulceration [2]. Since the 1990s, the global spread of STIs has significantly decreased, mainly thanks to public health interventions. However, AIDS, gonorrhoea, and syphilis, as the three major STIs, still cause a heavy disease burden worldwide.

Between 1990–2019, the age-standardised incidence of STIs worldwide showed a decreasing trend, but the number of cases and total disability-adjusted life-years of STIs increased globally, from 486.77 million in 1990 to 769.85 million in 2019 [3]. In the USA, the incidence of AIDS rose from 15.6 cases to 21.0 cases per 100 000 people between 2010–19 [4]. Between 1990–2019, the age-standardised incidence of gonorrhoea declined, and the age-standardised incidence of syphilis was relatively stable [3]. Therefore, the World Health Organization (WHO) proposed the global health sector strategies on, respectively, HIV, viral hepatitis, and STIs for the period 2022–30 to eliminate the public health hazards of STIs and advance universal health coverage, primary health care, and health security [5].

China has made significant progress in combating infectious diseases, and the overall burden of major infectious diseases has decreased substantially over time through the enforcement of legislation, as well as monitoring and screening for STIs [6]. By the end of 2022, there were 1.22 million HIV/AIDS patients in China. The number of cases of HIV infection in university students in China has been on the rise. Among all university students, 26.13% were sexually active, and nearly 35.00% of sexually active students had risky sexual behaviours [7]. Between 2004–21, a total of 2 289 435 cases of gonorrhoea have been reported in China [8]. The prevalence of gonorrhoea was 6.9% and 2.5% among female sex workers and men who have sex with men, who were high-risk populations [9]. People living with HIV (PLWH) were at an increased risk of syphilis. The overall prevalence of syphilis among PLWH in China was 19.9% [10]. Some studies had found that the seasonal characteristics of syphilis in China [11,12].

Previous studies have examined the general trend and the incidence and mortality among high-risk populations [9,13,14]. We aimed to explore the commonalities and differences by comprehensively comparing the trends and seasonality of three sexually transmitted infections in all provinces of the Chinese mainland between 2006–22, to provide reference for China to achieve the global health sector strategies on, respectively, HIV, viral hepatitis, and STIs for the period 2022–30.

## METHODS

### Data sources and collection

AIDS is characterised by Kaposi’s sarcoma and *Pneumocystis carinii* pneumonia, and is associated with innate immune dysfunction and decreased cluster of differentiation four T lymphocyte count. The progressive exhaustion of the immune system in AIDS patients allows life-threatening opportunistic infections and diseases to develop [15]. AIDS-related deaths refer to all deaths due to clinical diseases caused by AIDS, such as Kaposi’s sarcoma, *Pneumocystis carinii* pneumonia, and other opportunistic infections. AIDS, gonorrhoea, syphilis, and hepatitis A, B, and C were classified as class B infections in 1990, and other sexually transmitted infections were classified as class C infections. Given the data quality concern for diseases in class C, and the main route of hepatitis B transmission was mother-to-child transmission, and hepatitis C is mainly a bloodborne disease, we only focused on sexually transmitted diseases in class B [16].

The surveillance data of AIDS, gonorrhoea, and syphilis are more representative in China. After the 2003 severe acute respiratory syndrome epidemic, the updated China Information System for Disease Control and Prevention was also formally launched, providing a real-time and direct online reporting platform through the website to monitor infections. The China Information System for Disease Control and Prevention was a passive surveillance system. Hospitals, community service centres, and township health centres, based on the diagnostic criteria, reported and submitted information to the network system according to both clinical and laboratory diagnoses. Data on all notifiable infections are aggregated by the Chinese Centre for Disease Control and Prevention. Between 2006–22, the reporting standards and procedures of the system remained unchanged, and the long-term trend characteristics of the data were stable. Before using the data, we cross-checked the data collection protocols and procedures of the system to ensure that the data were collected in a standardised and scientific manner.

We obtained data on the number of cases and deaths of AIDS, gonorrhoea, and syphilis, stratified by year (2006–22), sex, age (<1, 1–14, 15–24, 25–44, 45–59, 60–74, ≥75 years), and province. In terms of data collection, we did not selectively choose data from specific periods or regions. By covering the entire mainland region, we aimed to represent the overall situation of sexually transmitted infections in China, thereby avoiding selection bias. In this study, Mainland China refers to 22 provinces, five autonomous regions, and four municipalities directly under the central government, totalling 31 provinces (excluding Hong Kong, Macau, and Taiwan). We extracted population estimates, including sex, age, and geographical area stratification from the National Bureau of Statistics of China, which had reported complete, accurate and authoritative data since 1952. For extreme values in the incidence and mortality that deviated significantly from the overall distribution, we examined the original data collection records to determine if they were due to data entry errors. If they were genuine but extreme, we retained them.

### Statistical analysis

We plotted trends in AIDS, gonorrhoea, and syphilis by sex and age using locally weighted regression. We used joinpoint regression analysis (Technical note S1 in the **Online Supplementary Document**) to detect changes in temporal trends and to estimate the average annual percentage change (AAPC) and corresponding 95% confidence interval (95% CI). We used the same model to test for the differences across sex and age subgroups. Joinpoint regression analysis identifies time points in which trends significantly change, using calendar year as the timescale. We also estimated the annual percentage change (APC) for each trend segment. APC>0 indicates an increase in incidence or mortality during the segment, and APC<0 indicates the opposite.

The Bayesian structural time series (BSTS) model (Technical note S2 in the **Online Supplementary Document**) has unique advantages in analysing epidemiological time series data, especially in seasonal decomposition, uncertainty quantification, and multi-component modelling [17]. BSTS employs a state-space framework to explicitly decompose time series into trend, seasonality, and regression components. This allows us to individually quantify the strength of seasonal effects and track dynamic changes in seasonality over time [18]. We used BSTS to fit the monthly incidence data of three types of notifiable STIs from January 2006 to December 2022. We obtained the estimates of seasonal component parameters based on the fitting results, using a 5000-iteration Markov Chain Monte Carlo algorithm. We compared the distribution of seasonal factors between 2006–22 to explore the consistency of each year. Then, we calculated the mean and 95% CI of seasonal factors to evaluate the seasonal trend of notifiable STIs. The month with a seasonal factor >0 was a high-incidence month, and the month with a seasonal factor <0 was a low-incidence month. National seasonality was considered to exist when high or low incidence months were present for more than three consecutive months. The same method was used to further compare the seasonal distribution of AIDS, gonorrhoea, and syphilis by province.

Spatial autocorrelation analysis encompasses both global spatial autocorrelation analysis and local spatial autocorrelation analysis (Technical Note S3 in the **Online Supplementary Document**). We used global spatial autocorrelation analysis to identify whether the spatial distribution of an attribute value in the whole study area is clustered. Further, we used local spatial autocorrelation analysis to estimate the degree of correlation between units and identify the type and location of disease unit clusters [19]. We used the local Moran I statistic to evaluate whether neighbouring observations in a data set are similar or dissimilar, which could be used to identify spatial clusters [20]. In this study, local Moran's I was used to identify spatial clusters of infections, which were further divided into hot spots (high-high clusters), cold spots (low-low clusters), and spatial outliers (low-high cluster and high-low clusters). Descriptive maps presenting the average yearly incidence and spatial cluster maps highlighting these four types of clusters were developed to depict the distribution of notifiable STIs. We selected four periods (2006–10, 2011–14, 2015–18 and 2019–22) to visualise the spatial and temporal dynamics of notifiable STIs. The incidence of each infection was divided into four classes by quantile classification and shown in different colours on the description map.

All analyses were done with *R*, version 4.4.0 (R Core Team, Vienna, Austria), Joinpoint Regression, version 5.0.2 (Statistical Methodology and Applications Branch, Surveillance Research Program, National Cancer Institute, Bethesda, Maryland, USA), and ArcGIS, version 10.8 (Esri, Redlands, California, USA). We considered *P*-value <0.05 as statistically significant.

## RESULTS

### Characteristics of the data

A total of 9 361 658 cases and 209 422 deaths of notifiable STIs were reported between 2006–22, resulting in an average yearly incidence of 40.15 cases per 100 000 and an average yearly mortality of 0.90 cases per 100 000 (**Table 1**). Syphilis had the highest average yearly incidence (28.75 cases per 100 000), and AIDS caused the highest average yearly mortality (0.89 cases per 100 000) and case-fatality (307.80 deaths per 1000 cases). Among three types of STIs, the average yearly case-fatality of the elderly aged ≥75 years was higher than other age groups, of which the average yearly case-fatality of AIDS is 589.37 deaths per 1000 cases.

**Table 1:** Incidence, mortality and case-fatality for notifiable STIs by gender and age groups, China, 2006–22

Characteristics	Total cases (n)	Yearly average incidence (per 100 000 population)	Total deaths (n)	Average annual mortality (per 10 000 000 population)	Case-fatality (per 1000 population)
Total	9 361 658	40.15	209 422	0.90	22.37
AIDS	677 167	2.90	208 432	0.89	307.80
Gender					
*Male*	517 438	4.33	163 367	1.37	315.72
*Female*	159 729	1.41	45 065	0.40	282.13
Age in years					
*<1*	49	0.02	18	0.007	367.35
*1–14*	4601	0.12	1476	0.04	320.80
*15–24*	42 237	1.36	8023	0.26	189.95
*25–44*	296 768	4.03	82 404	1.12	277.67
*45–59*	194 373	3.77	55 235	1.07	284.17
*60–74*	116 643	4.14	5931	1.66	400.50
*≥75*	22 458	2.47	13 236	1.46	589.37
Gonorrhoea	1 980 292	8.49	15	<0.001	0.008
Gender					
*Male*	1 621 804	13.57	11	<0.001	0.007
*Female*	358 488	3.15	4	<0.001	0.01
Age in years					
*<1*	8054	3.01	0	0	0
*1–14*	12 360	0.33	0	0	0
*15–24*	497 698	16.02	1	<0.001	0.002
*25–44*	1 095 707	14.88	4	<0.001	0.004
*45–59*	273 248	5.30	5	<0.001	0.02
*60–74*	2896	2.93	4	<0.001	0.05
*≥75*	10 654	1.17	1	<0.001	0.09
Syphilis	6 704 199	28.75	975	0.004	0.15
Gender					
*Male*	3 294 425	27.56	694	0.006	0.21
*Female*	3 409 774	30.01	281	0.002	0.08
Age in years					
*<1*	105 918	39.58	120	0.04	1.13
*1–14*	18 187	0.49	4	<0.001	0.22
*15–24*	813 366	26.19	23	0.001	0.03
*25–44*	2 579 642	35.04	192	0.003	0.07
*45–59*	1 505 076	29.18	228	0.004	0.15
*60–74*	1 122 884	39.85	172	0.006	0.15
*≥75*	559 117	61.55	236	0.03	0.42

### Trends in morbidity and mortality

**Figure 1:**
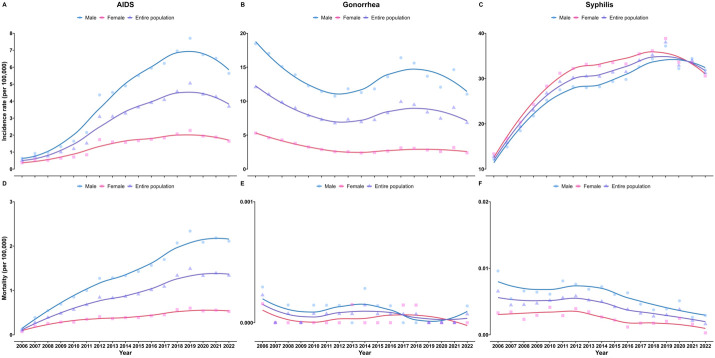
Incidence and mortality trend of notifiable STIs by sex, 2006–22. **Panel A.** The incidence of AIDS in the entire population and by gender. **Panel B.** The incidence of gonorrhoea in the entire population and different genders from 2006 to 2022. **Panel C.** The incidence of syphilis in the entire population and by gender. **Panel D.** The mortality of AIDS in the entire population and by gender. **Panel E.** The mortality of gonorrhoea in the entire population and by gender. **Panel F.** The mortality of syphilis in the entire population and by gender.

**Figure 2:**
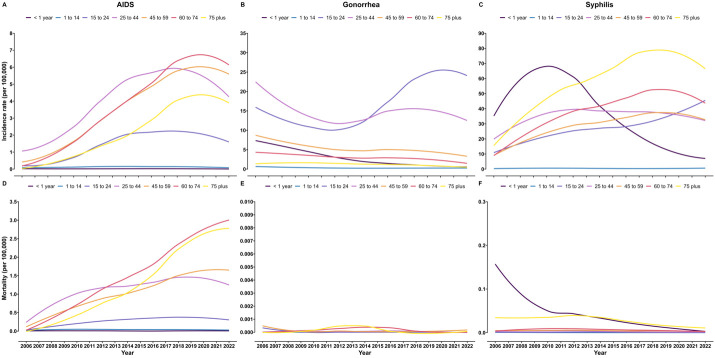
Incidence and mortality trend of notifiable STIs by age, 2006–22. **Panel A.** The incidence of AIDS. **Panel B.** The incidence of gonorrhoea. **Panel C.** The incidence of syphilis. **Panel D.** The mortality of AIDS. **Panel E.** The mortality of gonorrhoea. **Panel F.** The mortality of syphilis.

Between 2006–22, the incidence of notifiable STIs increased in adolescents, the elderly and males. The incidence of AIDS and syphilis increased between 2006–22, with AAPC = 14.66% (95% CI = 12.56, 16.94, *P* < 0.05) and AAPC = 5.69% (95% CI = 4.87, 6.43, *P* < 0.05). The cases and deaths of AIDS for males and females increased between 2006–22 (Table S1 in the **Online Supplementary Document**). The incidence of AIDS was higher in males than in females, and the increase rate of males was greater than females with AAPC = 15.11% (95% CI = 13.61, 16.97, *P* < 0.05) and AAPC = 11.29% (95% CI = 9.35, 13.30, *P* < 0.05) (**Figure 1**, Panel A; Table S2 in the **Online Supplementary Document**). The incidence of AIDS among individuals aged ≥15 years, especially the elderly, showed an increasing trend between 2006–22 (**Figure 2**, Panel A). The incidence of gonorrhoea was higher in males than in females, and both males and females showed a downward trend (**Figure 1**, Panel B). Increasing trends were observed for the incidence of gonorrhoea among those aged 15–24 years with AAPC = 2.51% (95% CI = 0.43, 4.19, *P* < 0.05), while other age groups decreased (**Figure 2**, Panel B; Table S2 in the **Online Supplementary Document**). The cases of syphilis for males and females increased between 2006–22 (Table S3 in the **Online Supplementary Document**). Different from AIDS and gonorrhoea, females showed a higher incidence of syphilis than males between 2006–20, and were overtaken by males between 2021–22 (**Figure 1**, Panel C). The incidence of syphilis among those aged 15–24 years (AAPC = 8.80%; 95% CI = 8.00, 9.82, *P* < 0.05), 60–74 years (AAPC = 9.08%; 95% CI = 8.10, 9.97, *P* < 0.05), and ≥75 years (AAPC = 8.37%; 95% CI = 7.57, 9.17, *P* < 0.05) increased (**Figure 2**, Panel C; Table S2 in the **Online Supplementary Document**).

The mortality of notifiable STIs was relatively high in the elderly. Only AIDS showed an increasing trend in mortality with AAPC = 16.75% (95% CI = 14.62, 18.92, *P* < 0.05). The mortality of gonorrhoea was relatively stable, and the mortality of syphilis showed a downward trend with an AAPC = –5.62% (95% CI = –8.04, –3.02, *P* < 0.05) (Table S2 in the **Online Supplementary Document**). The deaths of AIDS for males and females increased from 2006 to 2022 (Table S1 in the **Online Supplementary Document**). The increase in the mortality of notifiable STIs was mainly contributed by AIDS, and the increase rate of males was greater than females with AAPC = 20.03% (95% CI = 15.00, 25.29, *P* < 0.05) and AAPC = 13.42% (95% CI = 11.38, 16.12, *P* < 0.05) (**Figure 1**, Panel D, Table S2 in the **Online Supplementary Document**). Between 2006–22, the mortality of AIDS among individuals aged ≥15 years increased to varying degree, and by 2022, the mortality of those aged ≥60 years was higher than that of other age groups (**Figure 2**, Panel D). In China, the number of patients who died from gonorrhoea each year was no more than three, and the mortality remained at a relatively low level (**Figure 1**, Panel E, **Figure 2**, Panel E; Table S4 in the **Online Supplementary Document**). The deaths of syphilis for males and females increased, but the mortality showed a downward trend between 2006–22 (Table S3 in the **Online Supplementary Document**). The mortality of males was consistently higher than that of females (**Figure 1**, Panel F). A decreasing trend in the mortality of syphilis was observed among children aged <1 year between 2006–22, with an AAPC = –30.14% (95% CI = –49.06, –4.03, *P* < 0.05) (**Figure 2****,** Panel F; Table S2 in the **Online Supplementary Document**).

### Analysis of seasonality

**Figure 3:**
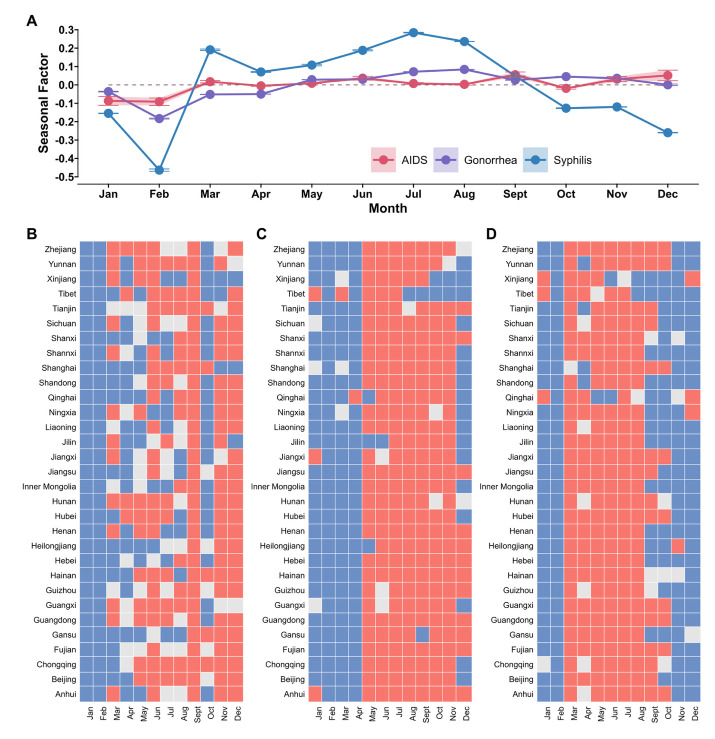
Seasonal trends of incidence of notifiable STIs in 31 provinces in China. **Panel A.** Seasonal trends of notifiable STIs. **Panel B.** Seasonal trend of AIDS, red represents the month of high incidence of AIDS (*P* < 0.05), blue represents the month of low incidence of AIDS (*P* < 0.05), and grey represents seasonal factors with no significant difference at 0 (*P* < 0.05). **Panel C.** Seasonal trend of gonorrhoea represents the month of high incidence of gonorrhoea (*P* < 0.05), blue represents the month of low incidence of gonorrhoea (*P* < 0.05), and grey represents seasonal factors with no significant difference at 0 (*P* < 0.05). **Panel D.** Seasonal trend of syphilis in 31 provinces of China, red represents the month of high incidence of syphilis (*P* < 0.05), blue represents the month of low incidence of syphilis (*P* < 0.05), and grey represents seasonal factors with no significant difference at 0 (*P* < 0.05).

The incidence of AIDS was not seasonal, while gonorrhoea and syphilis had seasonality in the Chinese mainland. The seasonal component of AIDS incidence in the Chinese mainland alternated between positive and negative values across months, with no continuity observed in the months with high incidence and those with low incidence. The seasonal factors of gonorrhoea (low incidence months: January–April, high incidence months: May–November; *P* < 0.05) and syphilis (low incidence months: October–February of the next year, high incidence months: March–September; *P* < 0.05) incidence in Chinese mainland had significant seasonal distribution (**Figure 3**, Panel A). Because of the high incidence of syphilis, the number of cases of syphilis in the high incidence month was far higher than that of AIDS and gonorrhoea. There were differences in the distribution of notifiable STIs among 31 provinces in the Chinese mainland. The seasonal factors of AIDS in most provinces fluctuated between high incidence and low incidence (**Figure 3**, Panel B). The seasonality of AIDS was unstable in most provinces, and the distribution of seasonal factors was constantly changing from 2006 to 2022 (Figure S1 in the **Online Supplementary Document**). The seasonal distribution of gonorrhoea and syphilis in 31 provinces was consistent with the seasonal distribution of the Chinese mainland, and the distribution trend of each province was similar. The high incidence months and low incidence months had a certain continuity (**Figure 3**, Panel C–D). The distribution of seasonal factors of gonorrhoea and syphilis in most provinces was consistent between 2006–22 (Figure S2–3 in the **Online Supplementary Document**).

### Spatial trend analysis

Between 2006–22, the incidence of AIDS, gonorrhoea, and syphilis and the case-fatality of AIDS gradually showed a trend of spatial clustering. The geographical range with a high average yearly incidence of AIDS is concentrated in the Southwest, and the incidence and mortality in many provinces showed an increasing trend between 2006–22 (Figure S4–6 in the **Online Supplementary Document**). The high average yearly incidence of gonorrhoea was primarily observed in East China and South China, with a decrease in Shanghai and Zhejiang between 2006–22 (Figure S5, Panel A in the **Online Supplementary Document**). The high average yearly incidence of syphilis was mainly in East China and the Northwest (Figure S6 in the **Online Supplementary Document**). Between 2006–22, the spatial autocorrelation of the incidence of AIDS, gonorrhoea, and syphilis changed from non-significant (AIDS *P* = 0.95, gonorrhoea *P* = 0.09, syphilis *P* = 0.09) to significant (AIDS *P* < 0.05, gonorrhoea *P* < 0.05, syphilis *P* < 0.05) positive spatial autocorrelation, and the global Moran I revealed a concentration tendency of high-incidence (Table S5 in the **Online Supplementary Document**). AIDS and gonorrhoea did not form a large range of high-high cluster areas between 2006–10, and there were demarcated high-high cluster areas and low-low cluster areas between 2019–22 (Figure S7 in the **Online Supplementary Document**). Only one high-high cluster area (Yunnan) of the incidence of AIDS was detected in Chinese mainland between 2006–10, the scope of high-high cluster areas expanded to the Southwest between 2015–22. Between 2006–10 and 2019–22, the high-high cluster areas of the incidence of gonorrhoea shifted from East China to South China. Tibet had become a high cluster area of the incidence of syphilis between 2015–18 and 2019–22. The spatial autocorrelation of the case-fatality of AIDS showed significant (*P* < 0.05) positive spatial autocorrelation as well since 2015. AIDS did not form a large range of high-high cluster areas in 2006–10, and there were demarcated high-high cluster areas and low-low cluster areas in 2015–22, and the scope of high-high cluster areas (Sichuan, Yunnan, Guizhou, Guangxi, Hunan, Chongqing, and Hainan) expanded to Southwest in 2015–22 (Figure S8 in the **Online Supplementary Document**).

## DISCUSSION

Although STIs can be largely preventable, they still cause a significant disease burden. The gonorrhoea incidence in children aged <1 year between 2006–22 and the syphilis incidence in children aged <1 year between 2010–22 decreased in China. This implied that China had made substantial progress by implementing the prevention of mother-to-child transmission. As early as 2003, the Chinese government launched the prevention of mother-to-child transmission of HIV, syphilis, and hepatitis B virus. In June 2010, the Ministry of Health issued the China 2010–20 action plan for syphilis control and prevention, indicating the national government’s commitment to controlling the mother-to-child transmission of syphilis. The policy set a goal of reducing congenital syphilis incidence to less than 15 cases per 100 000 live births by 2020 through the triple elimination of mother-to-child transmission of HIV, hepatitis, and syphilis [21]. In 2015, it began to achieve universal coverage. Research has shown that the proportion of pregnant women who initiated receiving syphilis treatment within 14 weeks of gestation increased from 12.4% in 2013 to 50.81% in 2019 [22]. We identified that the long-term trend of the incidence of STIs among individuals aged 15–24 years was growing between 2006–22, which may be caused by a series of contributing factors [23]. In China, with changes in attitudes towards sexual behaviour, approximately 60–80% of university students accept premarital sex and have multiple sexual partners. Coupled with the lack of sex education, the risk of transmission of STIs increased. Another factor was the use of social media among university students, which increased exposure to pornographic content and promoted convenient contact with various potential partners [23,24]. Based in schools and universities, adolescents need to be educated about sexual and reproductive health, including online peer education, psychological counselling and education, and HIV self-testing should be taken [25]. Schools could utilise anonymous consultation platforms, such as WeChat mini-programs, to incorporate interactive STI education into the compulsory curriculum, thereby reducing stigma-related delays in seeking care. It is worth noting that the incidence of AIDS and syphilis among the elderly in China had exceeded other age groups, and the global AIDS epidemic had a similar ageing trend. It is predicted that the number of people living with HIV aged ≥50 in some European countries will account for nearly 70% of the total people living with HIV by 2030 [26]. With the improvement of the living standard of residents in the Chinese mainland, the physical condition and sexual demand of elderly male individuals were still at a high level. Ageing did not necessarily limit elderly people, especially elderly men, from engaging in risky sexual behaviour [27]. However, due to the lack of sexual health education, elderly men possibly had a lower ability to perceive risks, leading to the frequent occurrence of high-risk sexual behaviours. Unprotected commercial sex is the main way for elderly men to transmit HIV [28]. The government may consider incorporating community-based integrated screening (*e.g.* syphilis, HIV rapid tests) into routine chronic disease management at primary care centres.

Many countries also reported different seasonality of gonorrhoea. This difference may be attributed to differences in geographical distribution, socio-economic status, medical facilities, climate conditions, and residents’ lifestyle. The seasonality of gonorrhoea was first reported in the USA as early as 1971, indicating that the incidence of gonorrhoea was highest in the third quarter and lowest in the first quarter, a finding similar to that of this study [29]. The number of gonorrhoea cases in India was highest in the second quarter and lowest in the fourth quarter, with the peak in the second quarter coinciding with the school summer holidays and harvest season celebrations [30]. The sociological factors of the high incidence of gonorrhoea in China in summer and autumn are probably related to the school summer holiday. During this period, adults had relaxed supervision of adolescents. Young people also may travel for recreational purposes during the summer to increase the risk of transmission [31]. In addition, with the increase in sexual activities, there was also more sexual intercourse without the use of condoms during the summer vacation [32]. One possible reason for the decrease in the willingness to use condoms could be the hot and humid climate, with the average monthly temperature exceeding 25°C in most parts of China from July to September, according to the National Climate Centre. The warm weather likely increased outdoor social activities, such as night markets and camping, which in turn increased the opportunities for sexual contact. The seasonality of syphilis may be closely related to the patterns of sexual activity of the population, and research on patterns of sexual activity in China is still needed. Studies have shown that human hormones have an endogenous rhythm, and hormonal changes caused by photoperiod and temperature changes in different seasons may also lead to an increase in unsafe sex in specific months. For example, sexual intercourse in adolescent female was most common in summer and autumn, and least frequent in winter [33]. There were also seasonal fluctuations in male testosterone levels, and could affect sexual activity. The annual spikes in male testosterone may be associated with increased sexual activity [34]. However, less research has focused on the relationship between the seasonality of gonorrhoea and the season itself (*i.e.* meteorological factors). The number of cases of gonorrhoea in China has been found to be positively correlated with temperature, relative humidity and precipitation from between 2006–19 [35]. More researches are needed to support the association between gonorrhoea and meteorological factors. This study also found that the high incidence of syphilis was concentrated from March to August. Between 2016–22, all counties in Zhejiang Province, China showed obvious seasonality and periodicity, with a peak from May to September [11]. Syphilis seasonality had also been reported in other countries. Surveillance data for Mexico between 2007–17 indicated that the highest number of acquired syphilis cases were detected between March and August [36]. Korea reported that the incidence of secondary syphilis in men peaks in the summer between 2011–19 [37]. Similar to gonorrhoea, the Chinese population appeared to be more active in summer than in winter, which was likely related to sociological factors and hormonal fluctuations. In China, workers from rural areas typically begin travelling to cities around March (after the Lunar New Year), and the large-scale rural-to-urban migration in China may exacerbate localised syphilis outbreaks [38]. The study showed that rural-to-urban migrants had a higher sexual risk, with 31% having multiple sexual partners [39]. A meta-analysis showed that the prevalence of STIs among the rural-to-urban migrants in China was significantly higher than among the general adult population, including AIDS, syphilis, and gonorrhoea [40]. Therefore, the government needs to carry out safe sex education and strengthen screening for notifiable STIs before summer among high-risk groups, including female sex workers, men who have sex with men, drug users, migrant workers and the elderly. Reducing the number of sexual partners and condom use are key factors in reducing the risk of STIs. It seems to have been pivotal to success in two countries heralded for reversing their STIs epidemics, Thailand and Uganda. Thailand launches ‘100% condom,’ a collaborative program between local authorities and all ‘sexual entertainment establishments,’ to reduce sexual transmission of STIs by ensuring condom use [41]. The intervention also resulted in a significant decrease in the proportion of Thai men engaging in commercial and other casual sex [42]. The A-B-C (abstinence, be faithful, use condom) HIV prevention strategy in Uganda encourages individuals to delay sexual intercourse, reduce casual sex, and increase the use of condoms. A 70% decline in HIV prevalence in Uganda since the early 1990s has been associated with a 60% reduction in casual sex [43]. The Chinese government could encourage the reduction of casual sex and the provision of easy access to condoms. In addition, this study found that the lowest incidence of AIDS, gonorrhoea, syphilis in February. The possible reason was that most people were reluctant to seek medical help during the Lunar New Year (January or February). The monitoring system probably experienced delays during the Lunar New Year, which also explained the sudden increase in the number of reported cases of HIV, gonorrhoea and syphilis in March. In 1988, WHO declared 1 December of each year as ‘World AIDS Day.’ The CDCs, schools and related organisations determined the theme of STI prevention and treatment around December each year, and promoted sex safety education activities to the public in China. It is suggested to implement interventions ahead of the summer, providing free condoms and testing reagents to the population, especially for high-risk groups, which may be conducive to the prevention of STIs. Sufficient quantities of penicillin (syphilis) and ceftriaxone (gonorrhoea) drugs should be stored in key provinces before June each year to meet the peak demand, and the remaining drugs are dispensed to primary health institutions for routine treatment to avoid expired waste after November.

Between 2006–22, the incidence of AIDS, gonorrhoea, and syphilis in the Chinese mainland gradually showed a trend of spatial clustering, mainly in Southwest, East China, and West China. In 1985, China’s first AIDS case was found in Yunnan, and the first indigenous case was reported in Yunnan in 1989. Southwest China borders Southeast Asia, which is the main drug-producing region in the world. Injecting drugs were related to the early spread of AIDS in the Southwest [44]. In the 1990s, the China Centre for Disease Control and Prevention launched the intervention project to promote safe sexual behaviour among commercial sex workers in Yunnan, leading to a decrease in AIDS cases. Studies have shown that Sichuan Province had the most significant influx of HIV/AIDS cases in China between 2016–18, and the cases mainly came from Guangdong, Chongqing, Zhejiang, Yunnan, and Fujian [45]. The incidence of gonorrhoea in East China was likely influenced by various sociodemographic factors. Urban areas with high household income were more likely to have a higher incidence of gonorrhoea. More disposable income and business trips also enabled men to afford sexual services. Extramarital infections were the most common source of STIs [46]. Due to the evolution of gender beliefs and behaviours, infections caused by extramarital sexual behaviour were constantly increasing [46]. The research has shown that the passenger mobility rate can explain the high incidence of gonorrhoea more than population density, which also indicates the floating population played an important role in the transmission of STIs [47]. Some less developed areas, such as Tibet, may have limited early diagnosis capabilities. These undetected cases leaded to the continuous spread of the virus, who were discovered with the increase of health investment [48]. The spatial distribution and clustering analysis of STIs are crucial for public health practices, and this study provides target provinces that should strengthen prevention and control measures. The Chinese government provided free antiretroviral treatment to AIDS patients, which could effectively extend their lives. In these provinces with high case-fatality, the management and relief of AIDS patients may be insufficient, which should also be paid attention to while controlling the incidence.

This study has several limitations. First, according to the regulations of the China Centre for Disease Control and Prevention, health institutions must report all notifiable cases of infections through a network system. However, the coverage of township-level health facilities in some provinces is low, especially in less developed provinces in western China and rural areas, which may lead to an underestimation of infections. Some patients did not seek medical services. The reported annual incidence and mortality may be underestimated in some provinces. Like another passive reporting system, the underreport may be a potential bias in reporting, which should be acknowledged. But it’s still representative. It should be kept in mind that the reasons for the low mortality, whether due to underreporting or the real situation, should be explored. On the other hand, the incubation period of AIDS is relatively long, and the actual time of HIV infection is not available from surveillance data. This temporal discrepancy may obscure underlying seasonal trends. To address this limitation, future studies should incorporate active surveillance and data tracing of infection. Second, the population data used to calculate the incidence and mortality is the annual average population of the Chinese mainland, which may not take into account the impact of population mobility. Finally, due to data availability, this study only analysed the spatiotemporal distribution pattern of reported cases of STIs at the provincial level. Future research can focus on the following provincial administrative units to describe the diversity of notifiable STIs in the Chinese mainland.

## CONCLUSIONS

This study shows that the incidence and mortality of notifiable STIs in Chinese mainland increased between 2006–22, with the rapid growth among young people, the elderly and males. The incidence of gonorrhoea and syphilis has significant seasonality in the Chinese mainland. The school can integrate interactive STIs education into compulsory curricula, leveraging anonymised counselling platforms (*e.g.* WeChat mini-programs) to reduce stigma-driven delays in care-seeking. The government may consider incorporating community-based integrated screening (*e.g.* syphilis, HIV rapid tests) into routine chronic disease management at primary care centres. The government needs to strengthen screening and carry out sex education on STIs among high-risk populations before summer. Sufficient quantities of penicillin (for syphilis) and ceftriaxone (for gonorrhoea) drugs should be stored in key provinces by June each year to meet the peak demand. The remaining drugs should be dispensed to primary health institutions for routine treatment to avoid expired waste after November. The government can focus on the Southwest, East China, and West China, where the incidence grew rapidly, to control the prevalence of STIs while paying attention to the management and education of AIDS patients.

## Additional material


Online Supplementary Document
