# Supplementary material for: Analysis of the trend of notifiable sexually transmitted infections in China between 2006–22
Source: J Glob Health. 2025 Jun 27;15:04175. doi: 10.7189/jogh.15.04175 (PMC12201936; doi:10.7189/jogh.15.04175)
Supplement: Online Supplementary Document [file jogh-15-04175-s001.pdf]

Supplement to: Xu Q, Zhang X, Zhao T, Cai X, Zhang S, Wang M, Lu Q, Cui F. Analysis of the trend of notifiable sexually transmitted infections in China between 2006–22. J Glob Health. 2025;15:04175.

Supplementary：Technical Note, Additional Figures and Tables

Technical Note S1. Joinpoint regression

Joinpoint regression analysis fits a series of joined linear models of the natural logarithm of annual incidence using calendar year as the independent variable, which is usually useful to describe the changes of the trend data. We use the log-linear model to analyze the trend of incidence and mortality of notifiable sexually transmitted infections (STIs) based on the population. The joinpoint regression model may be written as

$$E[y \mid x] = e^{\beta_0+\beta_1 x+\delta_1(x-\tau_1)^{+}+\cdots+\delta_k(x-\tau_k)^{+}}$$

where y is the outcome of interest, x is the calendar year, e is the natural base, β_0 is the constant coefficient, β_1 is the regression coefficient, k is the number of joinpoint, τ_k is the unknown joinpoint, δ_k is the regression coefficients of the k-th period segment.

The joinpoint regression model establishes all possible interval piecewise function connection points (i.e. Joinpoint points) using the grid search method (GSM), and calculates the sum of squares errors (SSE) and mean squared errors (MSE) corresponding to each possible scenario. The grid point with the smallest MSE is selected as the piecewise function connection point.

We allowed a maximum of 2 joinpoints for estimation as suggested by the program developers and used weighted bayesian information criterion (WBIC) to select the best-fitted model. Once the τ_k was determined, we estimate the annual percent of changes (APCs) of each period segment. We also estimated the average annual percent change (AAPC) assuming there is only one segment for the full range of our study periods. Joinpoint regression analysis was conducted with joinpoint Regression Program version 5.0.2 (Statistical Research and Applications Branch, Surveillance Research Program, Division of Cancer Control and Population Sciences, National Cancer Institute).

In this study, we noted that our incidence and mortality data contained some values of zero, and we substituted these zeros with 1% of the smallest incidence or mortality.

Technical Note S2. Bayesian structural time series models

The BSTS model is mainly consists of the Kalman filter, spike-and-slab method, and Bayesian model averaging, which enables it to consider time-varying model parameters at the same time and present the random characteristics of the target sequence. The prediction results of the BSTS method rarely depend on certain assumptions and can effectively deal with the uncertainties contained in the time series. The BSTS model was widely used in disease monitoring and prediction, causal inference, trend analysis, and policy evaluation and other fields. Many studies had found that the BSTS model has better prediction performance than other time series models. The equations of BSTS are as follows:

$$y_t = Z_t^T \alpha_t + \mu_t + \tau_t + \varepsilon_t \qquad \varepsilon_t \sim N(0, H_t) \qquad (1)$$

$$\alpha_{t+1} = T_t \alpha_t + R_t \eta_t \qquad \eta_t \sim N(0, Q_t) \qquad (2)$$

Equation (1) is the observation equation, while Equation (2) is referred to as the state equation. y_t is the expected incidence of notifiable STIs in month t. α_t is the potential d-dimensional state vector for month t. Z_t is defined as the d-dimensional output vector. T_t referred to as the transition matrix, is a $d \times d$ dimensional matrix that governs the evolution of the state vectore α_t over time. R_t is identified as the $d \times q$ control matrix, which, along with T_t, contains a mix of known values (0 and 1). ε_t and η_t are Gaussian error terms with mean 0 and variances H_t and Q_t. μ_t and τ_t are used to control for long-term trends and seasonal effects (12 month per year), respectively.

Technical Note S3. Formulas of global and local Moran's I

Two forms (global and local) of Moran's I statistic were adopted to explore the regional disparities in the burden of notifiable STIs among the 31 provincial units. Moran's I is a widely accepted measure for describing the spatial distributions of disease cases and for identifying unusual units or subsets of units. Global Moran's I is a measure for describing the overall spatial distribution characteristic of the entire area, while local Moran's I is the decomposition of global Moran's I for a particular area. That is, the sum of local Moran's I is proportional to global Moran's I. Global Moran's I varies between −1 and 1, while local Moran's I has no value limit. A positive value of global Moran's I indicates the infectious disease cases are clustered on a map, whereas a negative value implies the infectious disease cases are dispersed on a map. The described tendencies are more significant when the value approaches −1 and 1, whereas 0 indicates that the infectious disease cases are randomly dispersed.

The formulas of global and local Moran's I are defined as:

$$global\ Moran's\ I = \frac{n\sum_{i=1}^{n}\sum_{j=1}^{n}W_{ij}(y_i-\bar{y})(y_j-\bar{y})}{\sum_{i=1}^{n}\sum_{j=1}^{n}W_{ij}\sum_{i=1}^{n}(y_i-\bar{y})^2}$$

$$\text{local Moran}'s\ I = \frac{(y_i-\bar{y})}{m_0}\sum_{j}W_{ij}(y_j-\bar{y}) \quad m_0 = \sum_{i}(y_i-\bar{y})^2/n.$$

In the formula, n is the number of units in total, y_i and y_j are the incidence of a certain disease of units i and j, $\bar{y}$ is the average value. W stands for the row-standardized spatial weight matrix, W_{ij} is 1 if units i and j are adjacent and vice versa. The operation of summing over unit j is limited to the neighbors of unit i.

Table S1 Trend in the cases and deaths of AIDS in Chinese mainland from 2006-2022 by gender group

Year	Male			Female		
	Cases (n)	Deaths (n)	Case-fatality (per 1,000)	Cases (n)	Deaths (n)	Case-fatality (per 1,000)
2006	4 308	877	203.57	2 363	454	192.13
2007	6 289	2 648	421.05	3 438	1 256	365.33
2008	6 716	3 724	554.50	3 343	1 665	498.06
2009	9 095	4 742	521.39	4 186	1 854	442.90
2010	11 320	5 855	517.23	4 662	1 888	404.98
2011	14 909	6 916	463.88	5 541	2 308	416.53
2012	30 434	8 872	291.52	11 495	2 703	235.15
2013	31 638	9 003	284.56	10 648	2 434	228.59
2014	34 539	9 415	272.59	10 606	2 615	246.56
2015	39 005	10 116	259.35	11 325	2 639	233.02
2016	42 431	11 167	263.18	11 929	2 924	245.12
2017	44 612	12 163	272.64	12 582	3 088	245.43
2018	49 974	14 875	297.65	14 196	3 905	275.08
2019	55 511	16 848	303.51	15 693	4 151	264.51
2020	48 763	15 106	309.78	13 404	3 713	277.01
2021	47 170	15 792	334.79	12 984	3 831	295.06
2022	40 724	15 248	374.42	11 334	3 637	320.89

Table S2 Average annual percentage change in incidence and mortality of notifiable STIs from 2006 to 2022, China, by gender and age groups

	Incidence		Mortality	
	Trend	AAPC (95% CI)	Trend	AAPC (95% CI)
AIDS	Increase*	14.66% (12.56, 16.94)	Increase*	16.75% (14.62, 18.92)
Gender				
Male	Increase*	15.11% (13.61, 16.97)	Increase*	20.03% (15.00, 25.29)
Female	Increase*	11.29% (9.35, 13.30)	Increase*	13.42% (11.38, 16.12)
Age				
< 1†,‡	Stable	-15.04% (-32.21, 6.99)	Stable	-9.73% (-29.54, 15.76)
1-14	Stable	-0.49% (-4.65, 4.19)	Increase*	4.58% (1.90, 8.67)
15-24	Increase*	14.71% (11.39, 18.98)	Increase*	17.26% (13.81, 20.74)
25-44	Increase*	10.63% (8.46, 12.93)	Increase*	11.28% (9.35, 13.26)
45-59	Increase*	16.90% (15.62, 18.39)	Increase*	18.33% (16.05, 20.22)
60-74	Increase*	21.30% (19.78, 22.90)	Increase*	27.95% (25.71, 29.73)
⩾ 75	Increase*	26.29% (23.77, 28.68)	Increase*	32.64% (30.03, 35.37)
Gonorrhea‡	Decrease*	-3.18% (-4.24, -2.21)	Stable	-8.83% (-28.43, 16.30)
Gender				
Male‡	Decrease*	-2.71% (-4.17, -1.55)	Stable	-14.10% (-39.95, 22.99)
Female‡	Decrease*	-4.16% (-5.16, -3.28)	Stable	-2.40% (-20.41, 20.49)
Age				
< 1‡	Decrease*	-15.59% (-16.43, -14.73)	Stable	0.00% (0.00, 0.00)
1-14‡	Decrease*	-5.37% (-6.21, -4.52)	Stable	0.00% (0.00, 0.00)
15-24‡	Increase*	2.51% (0.43, 4.19)	Decrease*	-27.63% (-34.59, -8.37)
25-44‡	Decrease*	-3.34% (-4.90, -2.11)	Stable	-6.86% (-27.58, 20.79)
45-59‡	Decrease*	-5.62% (-6.56, -4.91)	Stable	1.00% (-22.76, 33.44)
60-74‡	Decrease*	-6.37% (-7.51, -5.45)	Stable	-3.49% (-26.59, 27.33)
⩾ 75‡	Decrease*	-6.06% (-7.22, -4.79)	Stable	-1.93% (-20.83, 21.90)
Syphilis	Increase*	5.69% (4.87, 6.43)	Decrease*	-5.62% (-8.04, -3.02)
Gender				
Male	Increase*	6.15% (5.27, 7.00)	Decrease*	-5.43% (-8.086, -1.69)
Female	Increase*	5.22% (4.64, 5.76)	Decrease*	-12.74% (-18.35, -5.93)
Age				
< 1‡	Decrease*	-10.30% (-11.09, -9.46)	Decrease*	-30.14% (-49.06, -4.03)
1-14‡	Increase*	3.39% (2.11, 4.78)	Decrease*	-19.64% (-33.44, -2.45)
15-24‡	Increase*	8.80% (8.00, 9.82)	Stable	-21.21% (-45.18, 13.46)
25-44	Increase*	2.63% (2.00, 3.21)	Increase*	37.41% (27.81, 54.48)
45-59	Increase*	7.46% (6.69, 8.17)	Stable	-2.75% (-6.36, 1.00)
60-74	Increase*	9.08% (8.10, 9.97)	Decrease*	-5.04% (-8.08, -1.57)
⩾ 75	Increase*	8.37% (7.57, 9.17)	Decrease*	-7.09% (-11.91, -1.79)

* $p < 0.05$

† When incidence contained zero, we substitute the zero with 1% of the smallest incidence.

‡ When mortality contained zero, we substitute the zero with 1% of the smallest mortality.

Table S3 Trend in the cases and deaths of syphilis in Chinese mainland from 2006-2022 by gender group

Year	Male			Female		
	Cases (n)	Deaths (n)	Case-fatality (per 1,000)	Cases (n)	Deaths (n)	Case-fatality (per 1,000)
2006	82 178	65	0.79	85 192	21	0.25
2007	101 410	37	0.36	107 374	22	0.20
2008	126 256	45	0.36	131 218	15	0.11
2009	149 106	44	0.30	157 275	19	0.12
2010	173 434	42	0.24	185 100	27	0.15
2011	190 222	56	0.29	204 960	19	0.09
2012	196 507	53	0.27	213 567	26	0.12
2013	196 083	48	0.24	220 893	23	0.10
2014	198 724	51	0.26	220 367	18	0.08
2015	207 770	43	0.21	226 204	15	0.07
2016	212 515	45	0.21	225 684	8	0.04
2017	233 456	33	0.14	242 404	12	0.05
2018	246 650	27	0.11	248 217	12	0.05
2019	267 930	28	0.10	267 889	14	0.05
2020	232 992	37	0.16	231 443	17	0.07
2021	248 903	19	0.08	231 117	11	0.05
2022	230 289	21	0.09	210 870	2	0.01

Table S4 Trend in the cases and deaths of gonorrhea in Chinese mainland from 2006-2022 by gender group

Year	Male			Female		
	Cases (n)	Deaths (n)	Case-fatality (per 1,000)	Cases (n)	Deaths (n)	Case-fatality (per 1,000)
2006	124 995	2	0.02	33 800	1	0.03
2007	115 954	0	0	29 644	0	0
2008	103 179	1	0.01	27 639	0	0
2009	95 315	0	0	24 509	0	0
2010	84 295	1	0.01	21 249	0	0
2011	78 987	1	0.01	18 967	0	0
2012	74 857	1	0.01	16 996	0	0
2013	82 838	0	0	17 101	1	0.06
2014	79 508	2	0.03	15 965	0	0
2015	83 696	1	0.01	16 549	0	0
2016	96 993	1	0.01	18 031	0	0
2017	117 535	0	0	21 320	1	0.05
2018	112 193	0	0	20 963	1	0.05
2019	98 513	0	0	19 425	0	0
2020	87 322	0	0	17 838	0	0
2021	105 902	0	0	21 901	0	0
2022	79 722	1	0.01	16 591	0	0

Table S5 Global autocorrelation of the incidence of notifiable STIs in Chinese mainland from 2006-2022

Year	AIDS		Gonorrhea		Syphilis		Case-fatality[†]	
	Moran's I	*p value*	Moran's I	*p value*	Moran's I	*p value*	Moran's I	*p value*
2006	-0.043	0.95	0.086	0.09	0.088	0.09	0.118	0.04*
2007	0.039	0.27	0.106	0.05	0.084	0.11	0.019	0.49
2008	0.091	0.06	0.137	0.02*	0.099	0.07	-0.063	0.70
2009	0.101	0.03*	0.164	0.01*	0.111	0.05	-0.020	0.86
2010	0.102	0.03*	0.178	< 0.01*	0.107	0.05	0.068	0.17
2011	0.119	0.01*	0.207	< 0.01*	0.077	0.14	0.104	0.07
2012	0.165	< 0.01*	0.209	< 0.01*	0.078	0.13	0.187	< 0.01*
2013	0.206	< 0.01*	0.230	< 0.01*	0.047	0.27	0.240	< 0.01*
2014	0.228	< 0.01*	0.214	< 0.01*	0.067	0.17	0.104	0.06
2015	0.263	< 0.01*	0.216	< 0.01*	0.108	0.04*	0.157	0.01*
2016	0.301	< 0.01*	0.244	< 0.01*	0.146	0.02*	0.169	< 0.01*
2017	0.315	< 0.01*	0.252	< 0.01*	0.153	0.01*	0.263	< 0.01*
2018	0.304	< 0.01*	0.260	< 0.01*	0.210	< 0.01*	0.229	< 0.01*
2019	0.314	< 0.01*	0.284	< 0.01*	0.309	< 0.01*	0.320	< 0.01*
2020	0.353	< 0.01*	0.326	< 0.01*	0.325	< 0.01*	0.386	< 0.01*
2021	0.349	< 0.01*	0.299	< 0.01*	0.316	< 0.01*	0.336	< 0.01*
2022	0.361	< 0.01*	0.295	< 0.01*	0.400	< 0.01*	0.338	< 0.01*

* $p < 0.05$

† The case-fatality of AIDS.

Table S6 APC in incidence of notifiable STIs from 2006 to 2022, by gender and age groups

	Period 1		Period 2		Period 3	
	Period	APC (95% CI)	Period	APC (95% CI)	Period	APC (95% CI)
AIDS	2006-2014*	29.07% (23.61, 36.98)	2014-2022	1.86% (-3.97, 6.32)	-	-
Gender						
Male	2006-2013*	33.30% (28.82, 42.89)	2013-2019*	9.15% (3.71, 24.68)	2019-2022	-9.09% (-20.73, 1.20)
Female	2006-2013*	24.17% (18.32, 33.67)	2013-2022	2.20% (-2.62, 5.74)	-	-
Age						
< 1[†]	2006-2022	-15.04% (-32.21, 6.99)	-	-	-	-
1-14	2006-2018*	5.85% (2.18, 23.74)	2018-2022*	-17.34% (-43.67, -1.67)	-	-
15-24	2006-2010	20.77% (-12.48, 45.30)	2010-2013	72.27% (-16.41, 99.06)	2013-2022	-2.09% (-11.74, 17.35)
25-44	2006-2014*	24.56% (19.04, 32.77)	2014-2022	-1.74% (-7.69, 2.78)	-	-
45-59	2006-2013*	34.65% (31.00, 41.05)	2013-2019*	10.06% (6.02, 25.39)	2019-2022	-5.17% (-16.83, 8.14)
60-74	2006-2012*	50.05% (44.79, 57.95)	2012-2019*	13.04% (9.55, 20.52)	2019-2022	-6.56% (-18.53, 2.70)
≥ 75	2006-2011*	65.19% (54.62, 81.76)	2011-2019*	20.96% (16.63, 26.83)	2019-2022	-9.43% (-26.78,1.99)
Gonorrhoea	2006-2012*	-9.74% (-13.92, -6.77)	2012-2018*	6.19% (2.99, 15.17)	2018-2022*	-6.35% (-15.47, -1.61)
Gender						
Male	2006-2012*	-9.36% (-18.08, -5.49)	2012-2017*	8.35% (2.99, 20.47)	2017-2022*	-4.90% (-14.16, -1.20)
Female	2006-2014*	-10.03% (-12.61, -7.91)	2014-2017	11.52% (-0.09, 16.68)	2017-2022	-3.17% (-11.23, 0.76)
Age						
< 1	2006-2022*	-15.59% (-16.43, -14.73)	-	-	-	-
1-14	2006-2013*	-13.87% (-16.68, -11.53)	2013-2022	1.81% (-0.04, 4.04)	-	-
15-24	2006-2012*	-8.57% (-17.28, -3.22)	2012-2019*	16.64% (12.30, 34.70)	2019-2022	-4.63% (-22.00, 5.63)
25-44	2006-2012*	-11.11% (-19.38, -7.02)	2012-2017*	8.35% (2.74, 21.36)	2017-2022*	-4.63% (-15.00, -0.63)
45-59	2006-2014*	-8.03% (-10.82, -6.44)	2014-2017	7.30% (-0.44, 11.74)	2017-2022*	-8.93% (-14.80, -6.30)

60-74	2006-2014*	-5.71% (-12.38, -4.02)	2014-2018	1.56% (-3.16, 8.84)	2018-2022*	-14.90% (-24.38, -10.28)
≥ 75	2006-2009*	7.80% (0.73, 23.38)	2009-2018*	-5.04% (-7.39, -3.22)	2018-2022*	-17.32% (-25.07, -13.13)
Syphilis	2006-2010*	20.83% (16.57, 25.01)	2010-2019*	2.98% (2.13, 4.52)	2019-2022*	-4.43% (-11.83, -0.76)
Gender						
Male	2006-2010*	19.94% (15.88, 25.81)	2010-2019*	3.42% (2.51, 6.47)	2019-2022	-2.49% (-9.98, 1.55)
Female	2006-2010*	21.66% (18.16, 24.26)	2010-2019*	2.56% (1.77, 3.46)	2019-2022*	-6.38% (-11.33, -3.18)
Age						
< 1	2006-2010*	19.93% (15.32, 26.29)	2010-2014*	-14.25% (-17.68, -2.15)	2014-2022*	-20.65% (-23.68, -19.49)
1-14	2006-2010*	18.87% (11.09, 28.98)	2010-2017*	-10.69% (-15.55, -7.67)	2017-2022*	13.49% (7.90, 22.35)
15-24	2006-2011*	16.33% (13.01, 21.69)	2011-2017	2.36% (-3.72, 4.77)	2017-2022*	9.48% (6.22, 18.90)
25-44	2006-2010*	16.70% (13.27, 19.98)	2010-2019	-0.41% (-1.09, 1.77)	2019-2022*	-5.38% (-10.61, -2.13)
45-59	2006-2010*	25.84% (21.02, 29.60)	2010-2019*	4.97% (4.01, 6.10)	2019-2022*	-6.58% (-11.87, -2.72)
60-74	2006-2010*	32.46% (26.18, 37.48)	2010-2019*	6.23% (5.05, 7.58)	2019-2022*	-8.84% (-14.81, -4.09)
≥ 75	2006-2010*	28.61% (23.61, 33.71)	2010-2019*	6.82% (5.55, 8.24)	2019-2022*	-9.93% (-15.96, -5.01)

* $p < 0.05$

† When incidence contained zero, we substitute the zero with 1% of the smallest incidence.

Table S7 APC in mortality of notifiable STIs from 2006 to 2022, by gender and age groups

	Period 1		Period 2		Period 3	
	Period	APC (95% CI)	Period	APC (95% CI)	Period	APC (95% CI)
AIDS	2006-2008*	104.51% (63.68, 134.19)	2008-2019*	10.83% (8.95, 15.15)	2019-2022	-2.75% (-17.06, 6.93)
Gender						
Male	2006-2008*	126.20% (57.48, 224.91)	2008-2022*	9.65% (7.80, 11.52)	-	-
Female	2006-2008*	88.66% (47.48, 132.04)	2008-2022*	5.46% (3.47, 7.12)	-	-
Age						
< 1†	2006-2022	-9.73% (-29.54, 15.76)	-	-	-	-
1-14	2006-2008*	82.59% (29.33, 156.65)	2008-2022*	-3.42% (-6.37, -1.30)	-	-
15-24	2006-2008*	166.51% (90.87, 237.52)	2008-2018*	9.93% (6.44, 16.66)	2018-2022	-8.59% (-28.56, 1.76)
25-44	2006-2008*	99.09% (58.74, 127.89)	2008-2019*	5.52% (3.89, 8.49)	2019-2022	-8.26% (-21.88, 0.47)
45-59	2006-2008*	88.08% (52.33, 119.43)	2008-2012*	20.56% (6.50, 30.29)	2012-2022	7.06% (-2.26, 10.65)
60-74	2006-2008*	130.46% (88.10, 165.29)	2008-2012*	38.26% (19.26, 52.64)	2012-2022*	10.28% (6.31, 12.67)
≥ 75	2006-2010*	101.81% (82.85, 131.66)	2010-2019*	21.26% (17.90, 29.98)	2019-2022	-0.80% (-18.06, 12.72)
Gonorrhoea†	2006-2022	-8.83% (-28.43, 16.30)	-	-	-	-
Gender						
Male	2006-2022	-14.10% (-39.95, 22.99)	-	-	-	-
Female	2006-2022	-2.40% (-20.41, 20.49)	-	-	-	-
Age						
< 1†	2006-2022	0.00% (0.00, 0.00)	-	-	-	-
1-14†	2006-2022	0.00% (0.00, 0.00)	-	-	-	-
15-24†	2006-2008*	-93.96% (-97.45, -41.35)	2008-2022	3.18% (-9.15, 34.87)	-	-
25-44†	2006-2022	-6.86% (-27.58, 20.79)	-	-	-	-
45-59†	2006-2022	1.00% (-22.76, 33.44)	-	-	-	-

60-74†	2006-2022	-3.49% (-26.59, 27.33)	-	-	-	-
≥ 75†	2006-2022	-1.93% (-20.83, 21.90)	-	-	-	-
Syphilis	2006-2013	0.68% (-4.52, 23.73)	2013-2022*	-10.25% (-23.44, -6.82)	-	-
Gender						
Male	2006-2014	-0.01% (-4.73, 31.90)	2014-2022*	-10.57% (-29.48, -6.17)	-	-
Female	2006-2020	-3.94% (-7.70, 10.84)	2020-2022*	-55.50% (-74.69, -14.51)	-	-
Age						
< 1†	2006-2022*	-30.14% (-49.06, -4.03)	-	-	-	-
1-14†	2006-2022*	-19.64% (-33.44, -2.45)	-	-	-	-
15-24†	2006-2022	-21.21% (-45.18, 13.46)	-	-	-	-
25-44	2006-2008*	2476.14% (594.05, 7073.72)	2008-2022*	-9.60% (-16.87, -3.15)	-	-
45-59	2006-2022	-2.75% (-6.36, 1.00)	-	-	-	-
60-74	2006-2009*	38.60% (15.68, 97.29)	2009-2020*	-6.16% (-9.19, -2.27)	2020-2022*	-42.51% (-55.48, -22.47)
≥ 75	2006-2014	1.75% (-5.06, 43.40)	2014-2022*	-15.15% (-37.50, -9.13)	-	-

* $p < 0.05$

† When mortality contained zero, we substitute the zero with 1% of the smallest mortality.

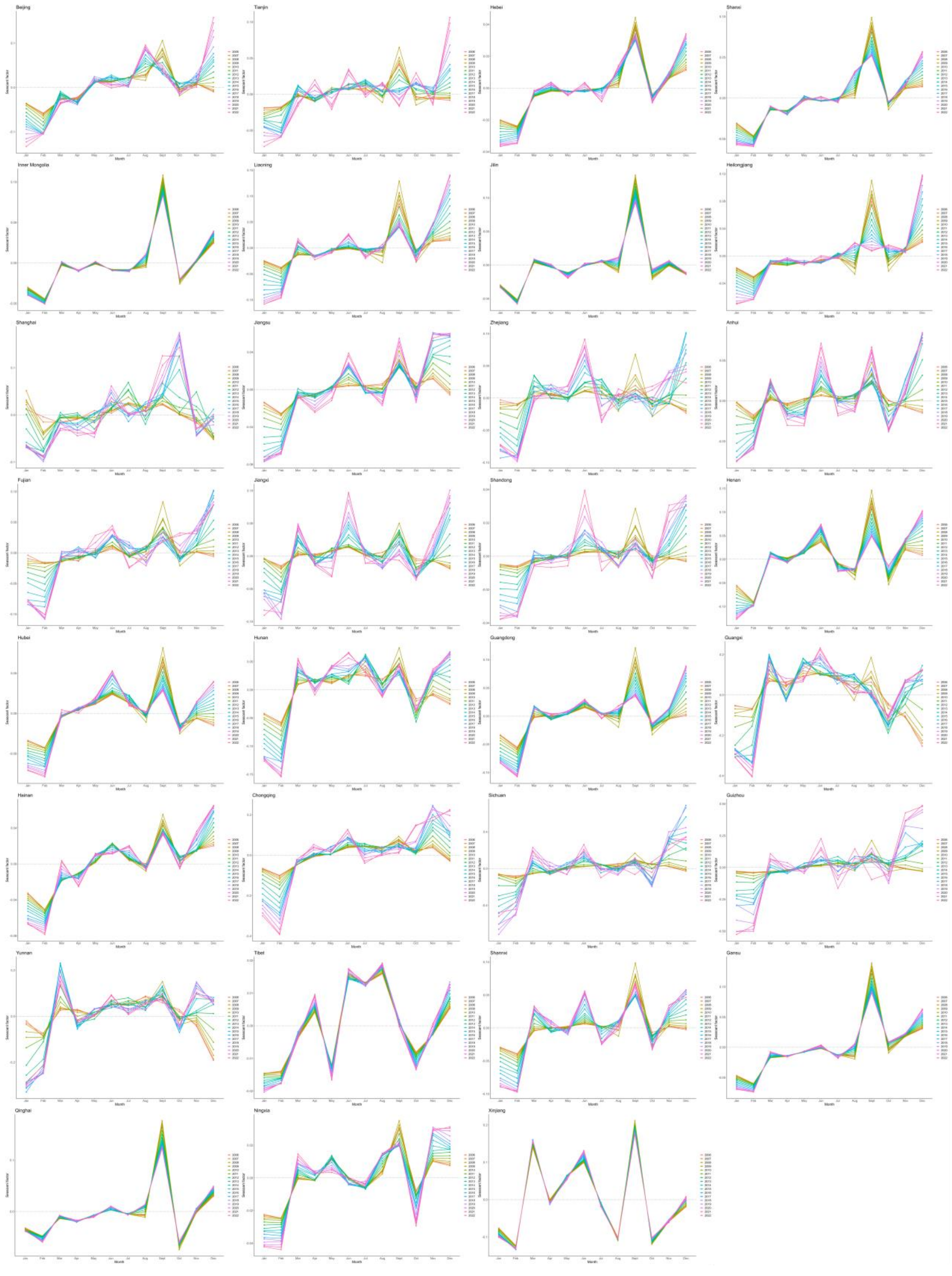

Figure S1 Seasonal factor distribution of AIDS incidence in 31 provinces of Chinese mainland from 2006 to 2022

* The figure comprised 31 charts, each representing the seasonal factor distribution of AIDS incidence in one of the 31 provinces in Chinese mainland from 2006 to 2022. Within each line chart, 17 lines are plotted, with each line corresponding to the annual seasonal factor pattern for a single year.

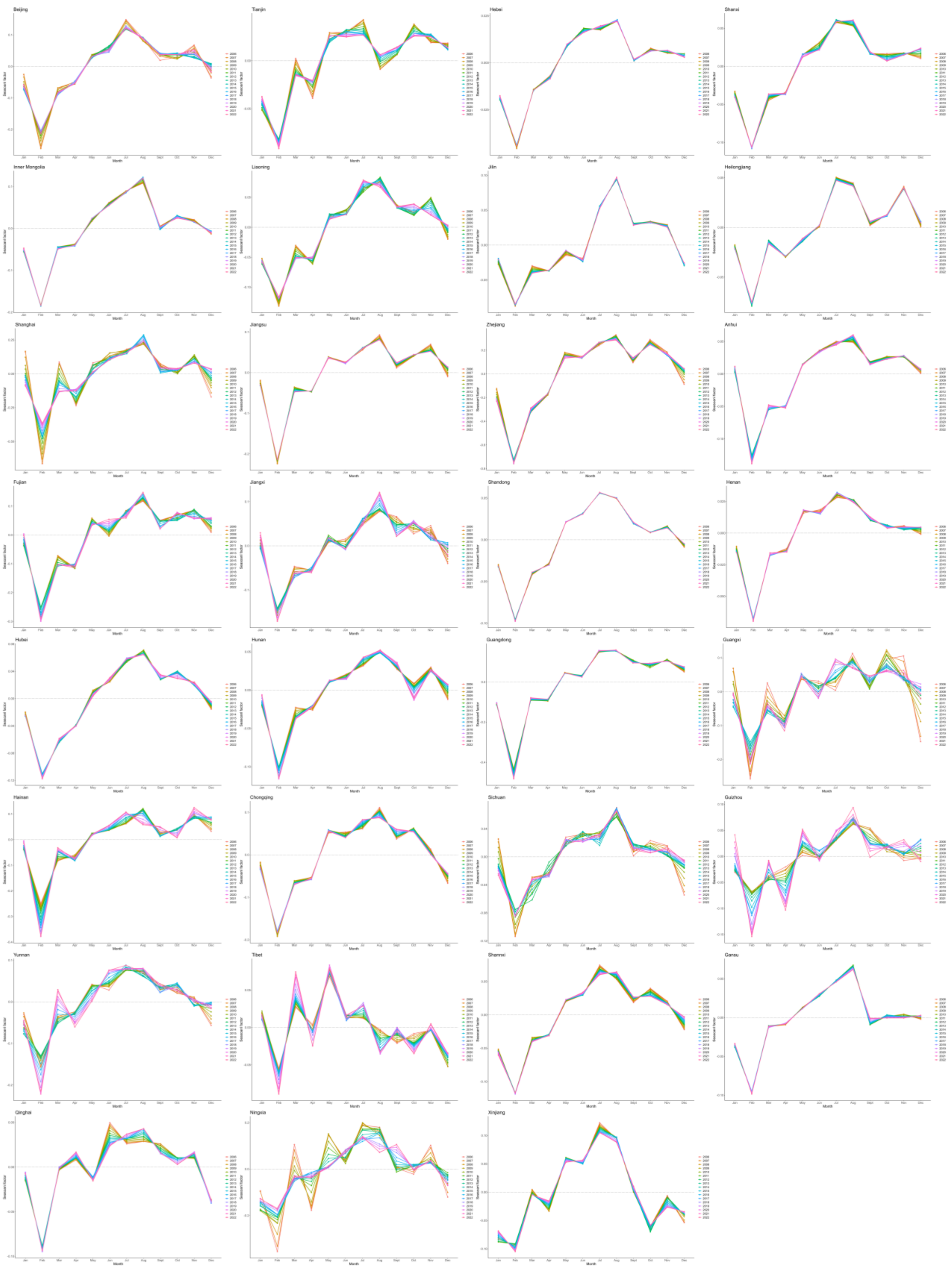

Figure S2 Seasonal factor distribution of gonorrhea incidence in 31 provinces of Chinese mainland from 2006 to 2022

* The figure comprised 31 charts, each representing the seasonal factor distribution of gonorrhea incidence in one of the 31 provinces in Chinese mainland from 2006 to 2022. Within each line chart, 17 lines are plotted, with each line corresponding to the annual seasonal factor pattern for a single year.

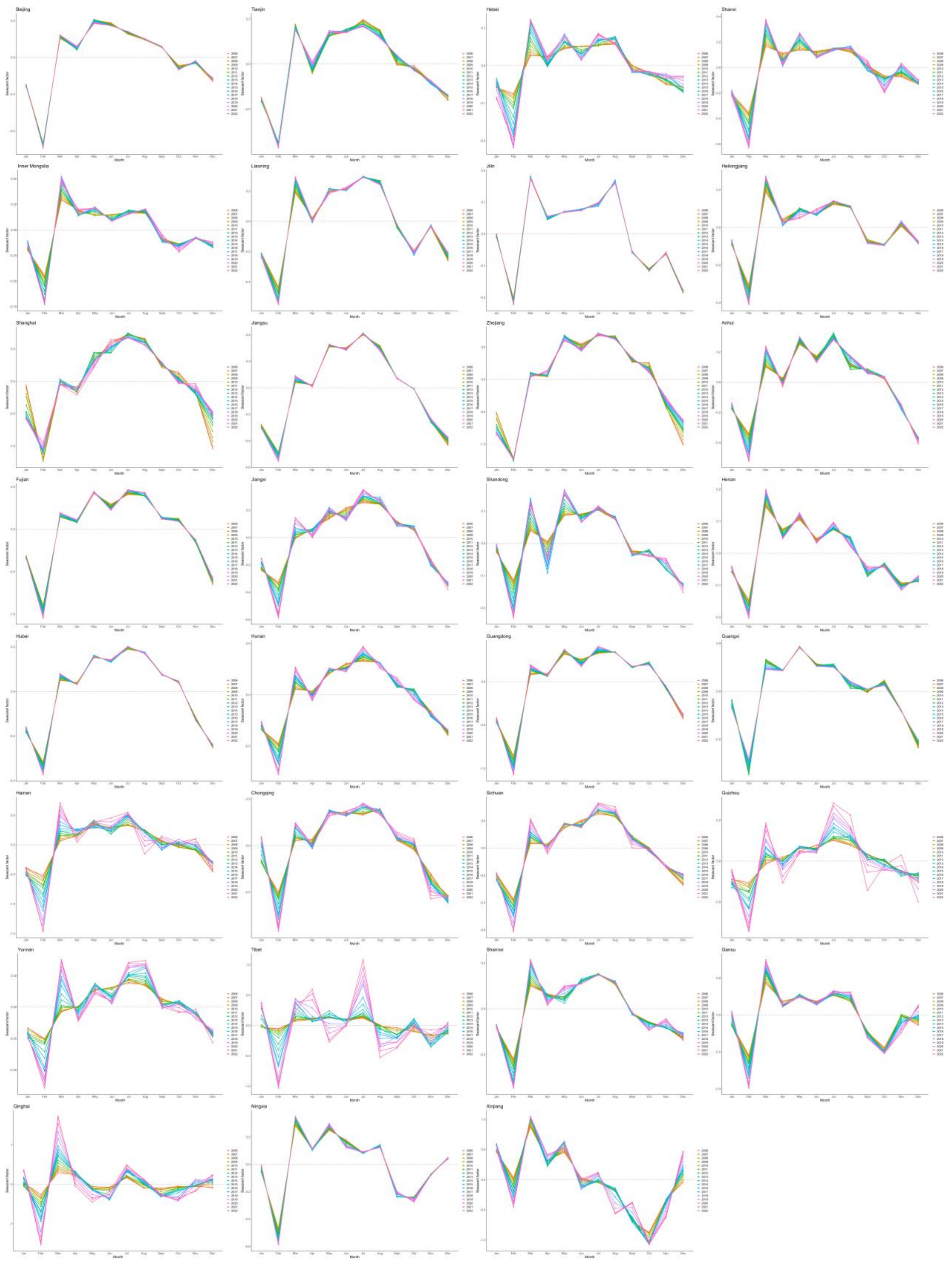

Figure S3 Seasonal factor distribution of syphilis incidence in 31 provinces of Chinese mainland from 2006 to 2022

* The figure comprised 31 charts, each representing the seasonal factor distribution of syphilis incidence in one of the 31 provinces in Chinese mainland from 2006 to 2022. Within each line chart, 17 lines are plotted, with each line corresponding to the annual seasonal factor pattern for a single year.

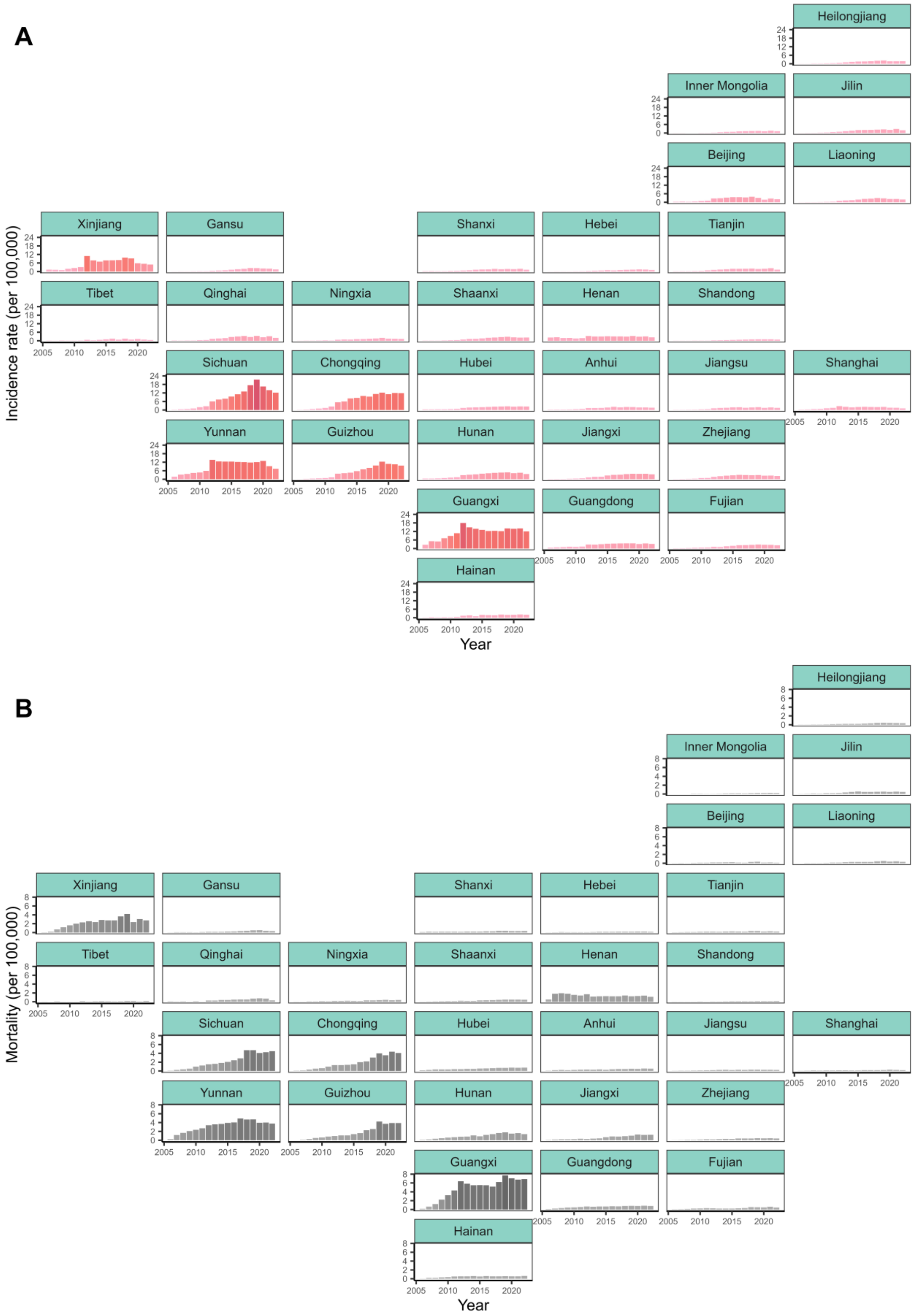

Figure S4 Incidence and mortality trends of AIDS in 31 provinces of Chinese mainland from 2006 to 2022

(A) The incident of AIDS in different provinces from 2006 to 2022, (B) The mortality of AIDS in different provinces from 2006 to 2022.

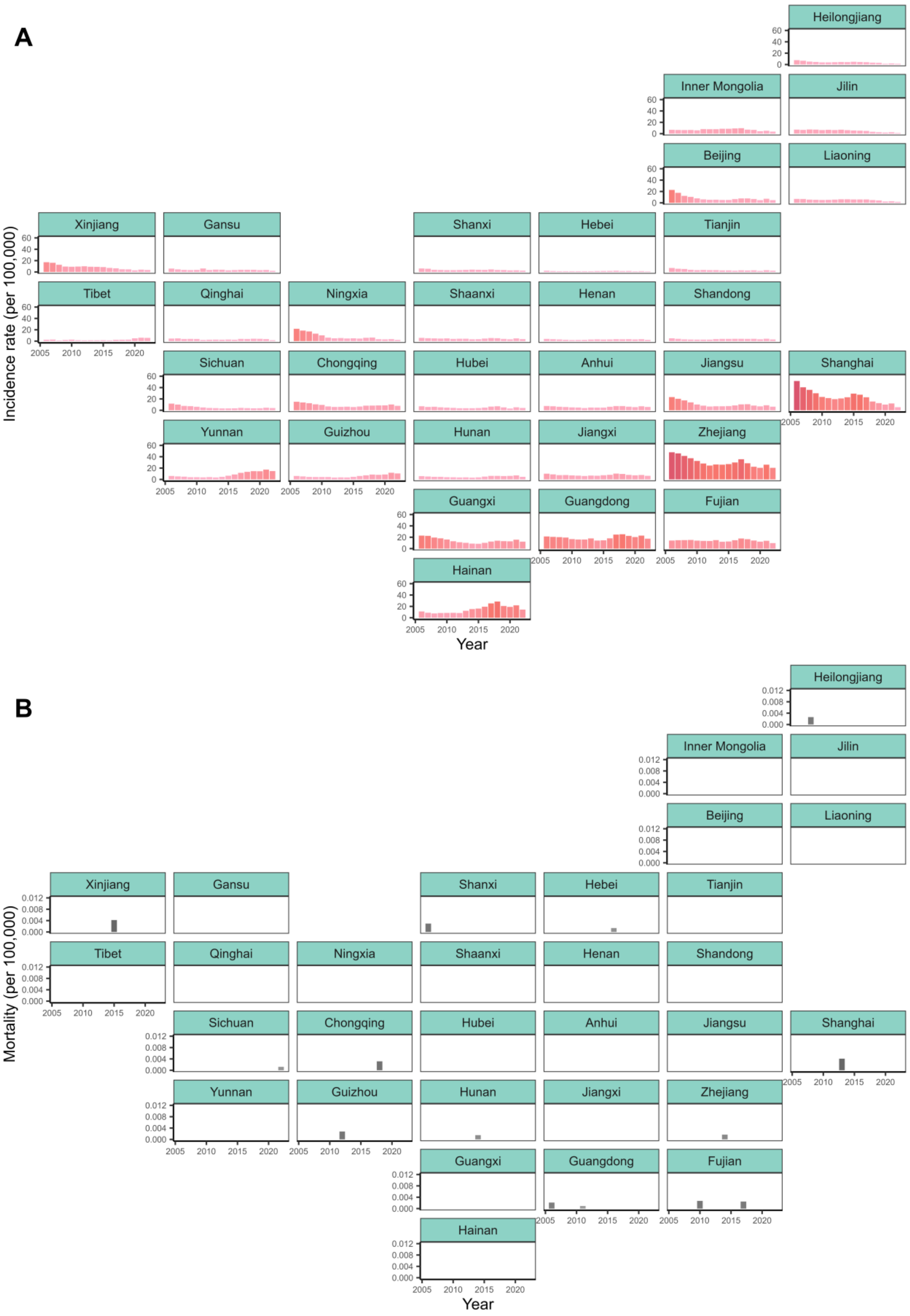

Figure S5 Incidence and mortality trends of gonorrhea in 31 provinces of Chinese mainland from 2006 to 2022

(A) The incident of gonorrhea in different provinces from 2006 to 2022, (B) The mortality of gonorrhea in different provinces from 2006 to 2022.

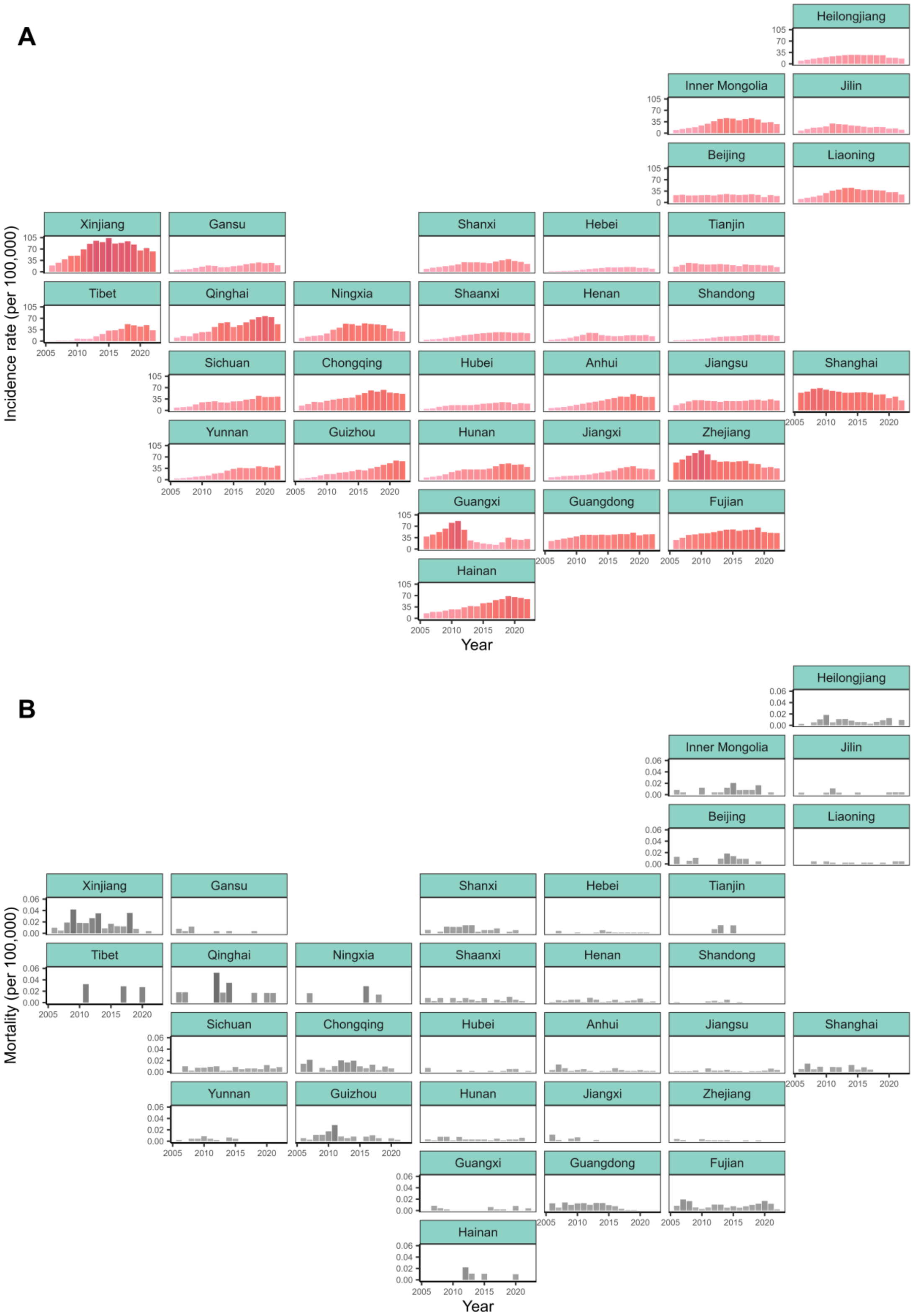

Figure S6 Incidence and mortality trends of syphilis in 31 provinces of Chinese mainland from 2006 to 2022

(A) The incident of syphilis in different provinces from 2006 to 2022, (B) The mortality of syphilis in different provinces from 2006 to 2022.

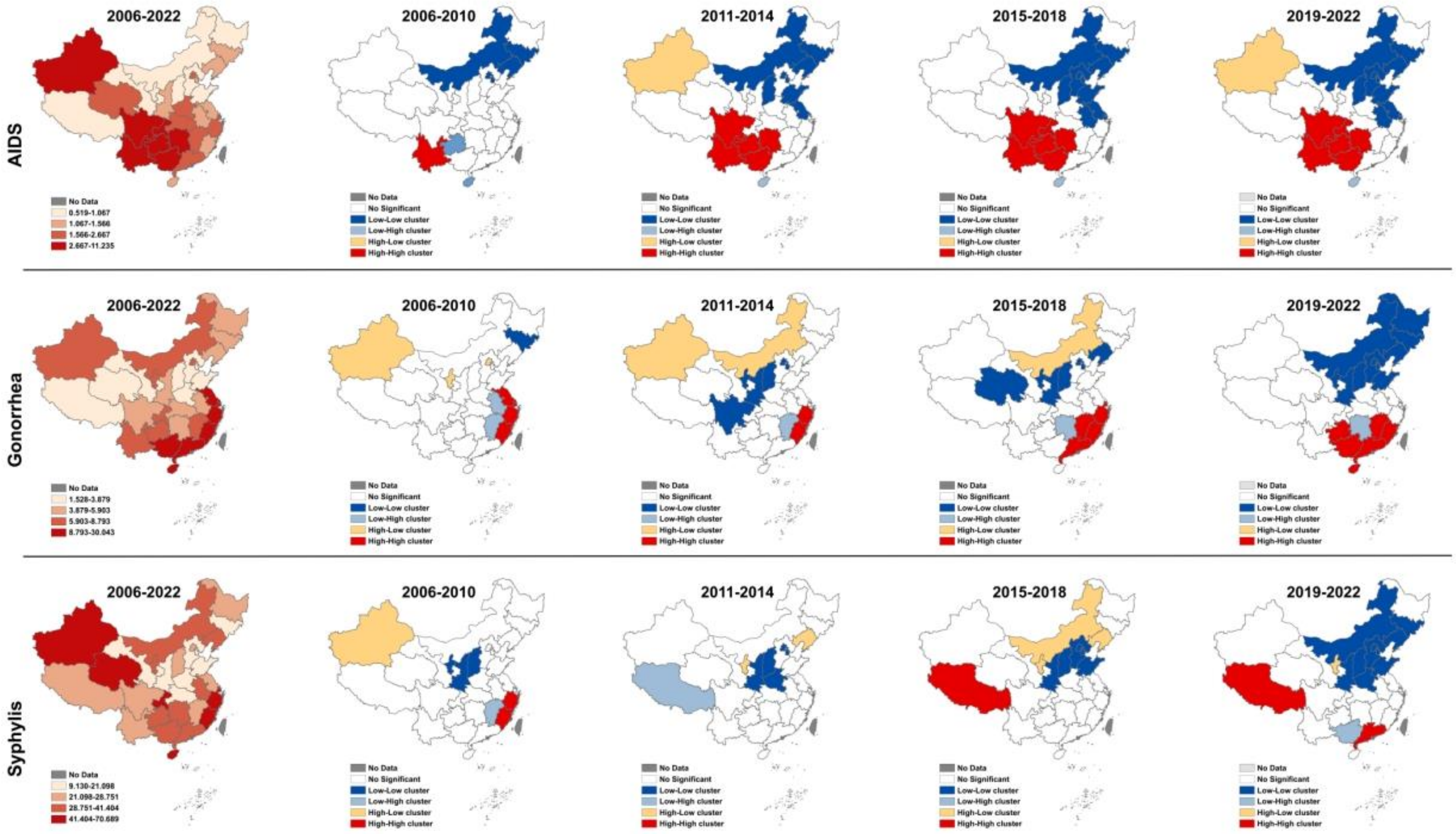

AIDS
2006-2022
2006-2010
2011-2014
2015-2018
2019-2022
No Data
0.519-1.067
1.067-1.566
1.566-2.667
2.667-11.235
No Data
No Significant
Low-Low cluster
Low-High cluster
High-Low cluster
High-High cluster
Gonorrhea
2006-2022
2006-2010
2011-2014
2015-2018
2019-2022
No Data
1.528-3.879
3.879-5.903
5.903-8.793
8.793-30.043
No Data
No Significant
Low-Low cluster
Low-High cluster
High-Low cluster
High-High cluster
Syphylis
2006-2022
2006-2010
2011-2014
2015-2018
2019-2022
No Data
9.130-21.098
21.098-28.751
28.751-41.404
41.404-70.689
No Data
No Significant
Low-Low cluster
Low-High cluster
High-Low cluster
High-High cluster

Figure S7 Descriptive maps and spatial cluster maps of incidence of notifiable STIs, 2006-2022

The first map in each row is a descriptive map, and the second to fifth maps are spatial cluster maps.

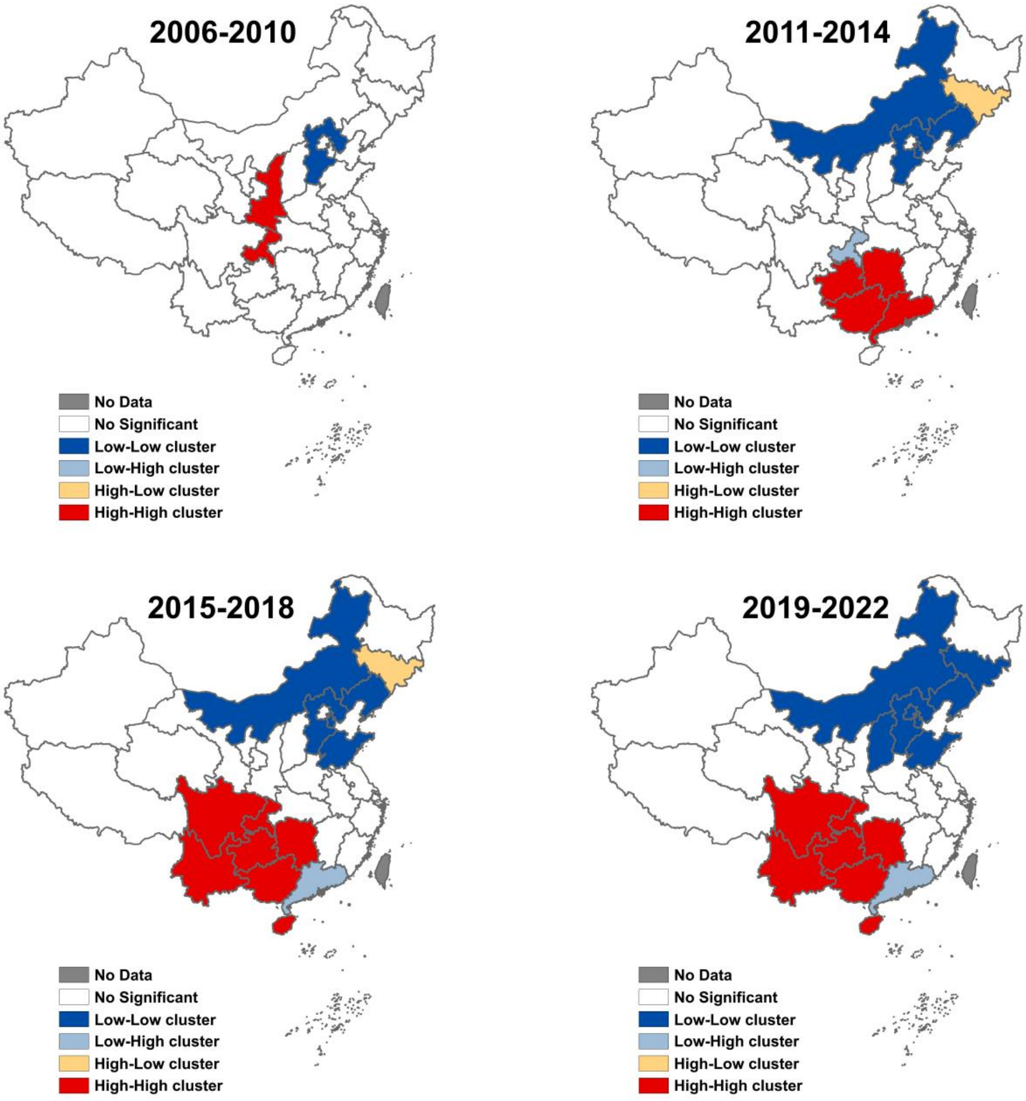

Figure S8 Spatial cluster maps of the case-fatality of AIDS in 2006-2010, 2011-2014, 2015-2018, and 2019-2022.
